# Bandwidth limits of luminescent solar concentrators as detectors in free-space optical communication systems

**DOI:** 10.1038/s41377-020-00444-y

**Published:** 2021-01-01

**Authors:** Mark Portnoi, Paul Anthony Haigh, Thomas J. Macdonald, Filip Ambroz, Ivan P. Parkin, Izzat Darwazeh, Ioannis Papakonstantinou

**Affiliations:** 1 Photonic Innovations Lab, Department of Electronic and Electrical Engineering, University College London, London, WC1E 7JE UK; 2 School of Engineering, University of Newcastle, Newcastle, NE1 7RU UK; 3 Department of Chemistry, Imperial College London, London, W12 0BZ UK; 4 Department of Chemistry, University College London, London, WC1H 0AJ UK; 5 Department of Electronic and Electrical Engineering, University College London, London, WC1E 7JE UK

**Keywords:** Solar energy and photovoltaic technology, Polymers

## Abstract

Luminescent solar concentrators (LSCs) have recently emerged as a promising receiver technology in free-space optical communications due to their inherent ability to collect light from a wide field-of-view and concentrate it into small areas, thus leading to high optical gains. Several high-speed communication systems integrating LSCs in their detector blocks have already been demonstrated, with the majority of efforts so far being devoted to maximising the received optical power and the system’s field-of-view. However, LSCs may pose a severe bottleneck on the bandwidth of such communication channels due to the comparably slow timescale of the fluorescence events involved, a situation further aggravated by the inherent reabsorption in these systems, and yet, an in-depth study into such dynamic effects remains absent in the field. To fill this gap, we have developed a comprehensive analytical solution that delineates the fundamental bandwidth limits of LSCs as optical detectors in arbitrary free-space optical links, and establishes their equivalence with simple RC low-pass electrical circuits. Furthermore, we demonstrate a time-domain Monte Carlo simulation platform, an indispensable tool in the multiparameter optimisation of LSC-based receiver systems. Our work offers vital insight into LSC system dynamic behaviour and paves the way to evaluate the technology for a wide range of applications, including visible light communications, high-speed video recording, and real-time biological imaging, to name a few.

## Optics: Optimising Luminescent Solar Concentrators for applications in optical systems

Modelling the bandwidth limits of Luminescent Solar Concentrators (LSCs) offers vital insights into their behaviour and provides a technique for evaluating their use in various applications. Although LSCs have emerged as a promising receiver technology in free-space optical communications due to their ability to collect light from a wide field-of-view and concentrate it into small areas, the comparably slow timescale of their fluorescence events may pose a bottleneck on the bandwidth of high-speed communication systems. A team of researchers led by Ioannis Papakonstantinou from University College London has developed a technique that delineates the fundamental limits of LSCs as optical detectors and demonstrated time-domain Monte-Carlo simulation for optimising LSC based receiver systems. The work opens the door for the use of LSCs in applications in visible light communications, high-speed video recording, and real-time biological imaging.

## Introduction

**Fig. 1:**
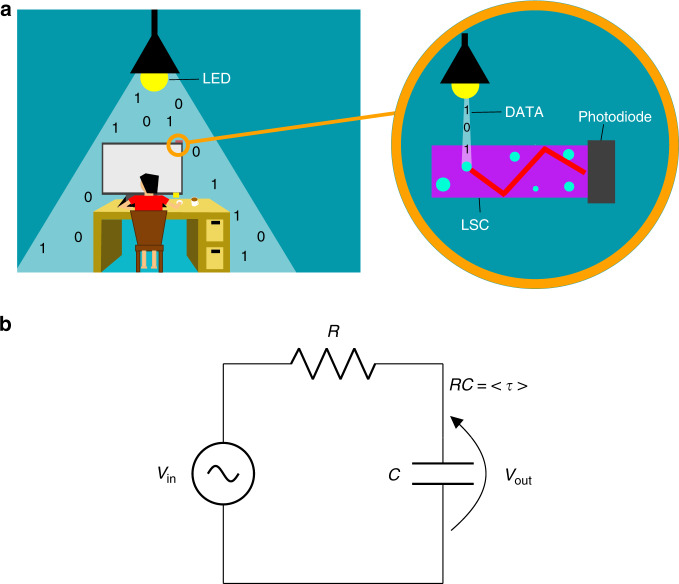
Overview of luminescent solar concentrators for optical communications. **a** Graphic depicting the principle of operation of an LSC-VLC system. Data are transmitted by modulating a source of light, typically LEDs. Light may come from any direction and is collected by fluorescent collectors that concentrate it to a photodiode on their edge. The photodiode converts the optical signal into an electrical signal, which is then processed by the receiver circuits. **b** At their limits, LSCs can be represented by a simple RC circuit, where *V*_in_ is the input and represents the absorbed incident light

Luminescent solar concentrators (LSCs) were originally proposed for the efficient collection of solar energy^1^ and have since found a prominent place in building integrated photovoltaic research^2–6^. In its simplest form, an LSC device is composed of a transparent host matrix doped with fluorescent materials (also known as fluorophores). The fluorophores absorb incident light and re-emit it in all directions at a longer wavelength, a phenomenon known as the Stokes shift. This re-emitted light is then trapped within the host matrix by means of total internal reflection, where it is waveguided to the edges of the device. In conventional optics, there is a trade-off between the maximum concentration gain and acceptance angle, known as conservation of étendue. This principle states that in the case of optical systems where the wavelength of light does not change, the maximum concentration gain, *C*_max_, is related to the refractive index of the concentrator, *n*, and the acceptance angle *θ*_a_ by *C*_max_ = *n*^2^*/*sin^2^*(θ*_a_*)*. However, étendue need not be conserved when Stokes shift is present, so the devices can simultaneously achieve high-concentration gain and a wide field-of-view, without violating the second law of thermodynamics^7,8^. Apart from photovoltaics, LSCs have attracted considerable interest for a diverse range of applications, including dark-field imaging^9^, microreactors^10^, greenhouse coatings^11^, and even dynamic systems (where the time of photon arrival is of essence), such as image recording and movement detection technologies^12,13^ and free-space optical communications. In the latter case, LSCs were introduced as an efficient means to collect the diffuse light generated by modulated light-emitting diodes (LEDs) in visible light communication (VLC) systems^14^, a concept serving as the paradigm in this paper and which is depicted in Fig. 1a.

The first demonstrations of LSCs for VLC predominantly focused their attention on how to increase their optical gain (optical power out at edge/optical incident power) and field-of-view (range of solid angles for which the detector accepts light). Planar geometries were initially considered^14^ before branching into more complex structures, such as parabolic geometries^15^. Following this, a dye-doped planar fluorescent collector was demonstrated with a field-of-view of 60° and boasting data rates of 190 Mb/s (ref. ^16^). A more complex ball-shaped fibre-based structure was subsequently demonstrated, exhibiting aggregated data rates of 2.1 Gb/s and a nearly complete field-of-view of 3.9*π* st (ref. ^17^). An alternative system combined the fluorescent layer with conventional focusing optics^18^, while further designs included flexible nanostructured devices^19^, fibre-based devices^20^, and organic–inorganic hybrids^21^. Finally, further enhancement in data rates was achieved in a multilayer device combining fluorescent materials designed to absorb different wavelengths in a wavelength division multiplexing arrangement^22^.

While the aforementioned examples probed the LSC optimisation problem on optical power and field-of-view grounds, little attention has been devoted to the performance of such devices in the time and frequency domains. Typically, fluorophore lifetimes are between 1 and 100 ns (refs. ^23,24^), which is orders of magnitude slower than the response time of currently employed high-quality communications semiconductor components, indicating that LSCs likely form the weakest link in optical communication channels. Worse still, the overlap between the absorption and emission spectra of fluorophores causes reabsorption, inducing further temporal delays and adding to the bandwidth (BW) limitations. In spite of LSCs potentially acting as barriers to high data-rate transmission, there is no existing study into the profound question of bandwidth limits in optical systems employing them.

This study makes a threefold contribution. First, we present a general analytical model for the prediction of the impulse response in arbitrary LSC geometries and show that for a large class of LSCs, for which reabsorption can be ignored, these LSCs behave as simple low-pass, RC circuits, as shown in Fig. 1b. Here, the effective fluorescence lifetime 〈*τ*〉 (which for the case of single-exponential decay distributions corresponds to the actual fluorescence lifetime) becomes the equivalent time constant of the circuit and, as is shown, is related to the device bandwidth, BW, via the very simple Eq. (1) (where the equality should be considered in this case).

1 $${\mathrm{BW}}\,({\mathrm{GHz}}) \le \frac{1}{{2\pi \left\langle \tau \right\rangle\, ({\mathrm{ns}})}}$$

Crucially, we subsequently prove that in the case of optically dense LSCs, for which multiple reabsorption events occur, the above equation becomes a strict upper bound and hence sets the fundamental bandwidth limit for any LSC integrated optical communication channel. In this case, we proceed one step further and resolve the impact of every additional reabsorption event on the system’s impulse response.

The second contribution of this paper is the development of a powerful time-domain, Monte Carlo (MC) modelling framework for LSCs. Unlike the analytical solution, which focuses solely on the system’s impulse response, the MC model can, in addition, predict the received power and the system’s field-of-view and hence infer fundamental quantities, such as the signal-to-noise ratio (SNR) and the bit-error ratio of the link. Furthermore, the MC algorithm can be deployed in more intricate optimisation scenarios where the exact geometry of the LSC (single layer or multilayer, planar or more complex 3D geometries), fluorophore properties, such as absorption and emission spectra, concentration and quantum yield (QY, Φ), salient properties of the light emitters and receivers (emission spectrum of LEDs, angular distribution of emitted fields, etc.) and environment (presence of noise, or multipath cross-talk) can be accounted for and incorporated into comprehensive multiparameter simulations.

Third, we highlight a subtle trade-off between maximising the collection efficiency of LSCs and increasing their bandwidth, accentuating the importance of evaluating LSC dynamic performance when employed for use in optical communication systems.

This paper is organised as follows: we start by introducing the time-domain MC algorithm and proceed to its experimental verification. Building on these results, we present our analytical solution for the BW of both tenuous and optically dense LSCs, and conclude by assessing the communication system performance in terms of throughput and *Q*-factor.

## Results

### Time-domain Monte Carlo ray tracing

MC ray tracing is the most popular simulation algorithm in static LSC research and is widely used to optimise light concentration from incoherent illumination sources, such as the Sun^2–5,25^. It is a versatile tool that, in addition to predicting the performance of conventional planar LSCs, has been used in the past to model more complex mechanisms, such as flexibility^26^, Forster energy transfer^27^, dichroic dye alignment^28^, and plasmonics^29^. In this regard, migrating MC techniques into the realm of dynamic LSC research seems a natural extension. In fact, time-domain methods have already been employed in conjunction with MC ray tracing for applications, such as medical imaging^30^ and even optical communications^31^; however, we believe this to be the first application of time-domain modelling to LSCs. A brief description of the modelling techniques used in this work can be found in the “Methods” section.

Standard LSC MC algorithms receive the fluorophore properties (absorbance, emission and absorption spectra, concentration, and quantum yield), host matrix properties (refractive index and geometry), and light source properties (spectrum and angular distribution of photons) as inputs and output the number of photons propagating at the edges of the LSC. In this work, an additional timestamp is associated with each photon, recording the cumulative time of all events occurring between the generation and detection of this photon. Simply put, when a photon of wavelength *λ* travels a distance *x* through a material with refractive index *n*(*λ*), the timestamp progresses by *t* = *xn*(*λ*)/*c*. This is cumulatively summed for photon propagation in all directions within the device. Upon absorption and re-emission of the photon, the timestamp further increases by an amount proportional to the fluorescence decay characteristics. The timestamp continues to increase, accounting for all multiple fluorescence and propagation events until the photon arrives at the desired LSC edge.

The time-dependent intensity, *I*(*t*), of a decay transition from an excited electronic state is given by Eq. (2) (ref. ^32^).

2 $$I\left( t \right) = I_0e^{ - \frac{t}{\tau }}$$

where a single-exponential component is assumed here with *I*_0_ the intensity at *t* = 0 and *τ* the inverse of the decay rate (fluorescence lifetime). In the case where multiple decay paths of different rates exist, the intensity is alternatively given by Eq. (3).

3 $$I(t) = \mathop {\sum }\limits_{m = 1}^M A_me^{ - \frac{t}{{\tau _m}}}$$

where *τ*_*m*_ is the decay rate of each of the *M* total possible paths and where *A*_*m*_ is a constant of proportionality, denoting the relative amplitudes of each transition. Such decay rates (single or multipath) can easily be characterised by time-correlated single photon counting (TCSPC)^25^, as shown in the Supplementary Material and Fig. S2.

To establish the required progression of the timestamps after each fluorescence event, inverse transform sampling is required. In this process, the intensity functions described in Eqs. (2) or (3) are interpreted as probability density functions (pdfs) and transformed so that a random number generator can be used to determine the delay introduced by a fluorescent event. In the case of a simple single exponential, as described in Eq. (2), this can be easily done analytically. The resultant inverse function *t*(*X*) is given in Eq. (4), where *X* is a uniformly generated random number between 0 and 1.

4 $$t\left( X \right) = - \tau {\mathrm{ln}}\left( {1 - X} \right)$$

In the case of more complex multiple exponential decays, such as that shown in Eq. (3), inverse sampling can be performed numerically. Upon assigning timestamp progressions to both fluorescence and propagation of the photons through an LSC, the impulse response of the devices can be simulated for any arbitrary LSC environment, which can in turn be used to predict LSC bandwidth behaviour.

### Experimental verification of the MC time-domain model

**Fig. 2:**
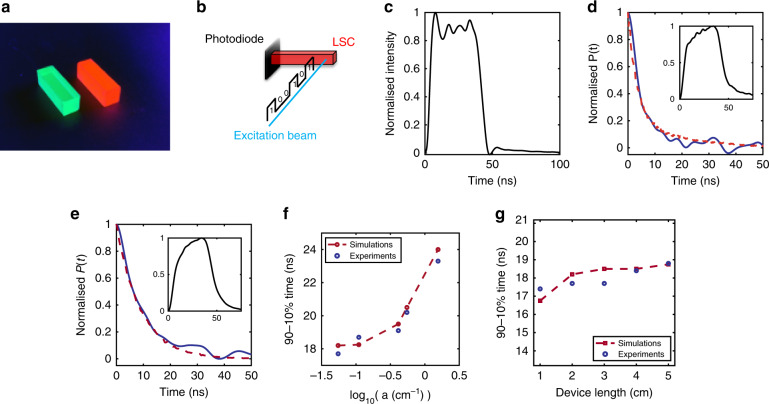
Impulse response prediction and measurement. **a** Photograph of Lumogen Red 305- and CsPbBr_3_-doped LSCs (0.67 × 0.67 × 2cm) under illumination by an ultraviolet lamp. **b** Diagram showing the experimental setup used to verify the Monte Carlo model. The excitation beam is perpendicular to the long axis of the LSC devices and parallel to the plane of the photodiode. **c** Time-domain pulse shape of the laser beam directly illuminating the photodiode. **d** Comparison between simulated (red dashed line) and experimentally derived (blue solid line) impulse responses for a “fast” CsPbBr_3_-doped LSC (0.67 × 0.67 × 2cm, *a* = 0.6cm^−1^). Inset: time-domain pulse shape of the light at the photodiode having passed through the corresponding LSC. **e** Comparison between simulated (red dashed line) and experimentally derived (blue solid line) impulse responses for “slow” Lumogen Red 305-doped LSCs (0.67 × 0.67 × 2cm, *a* = 0.5cm^−1^). Inset: time-domain pulse shape of the light at the photodiode having passed through the corresponding LSC. **f** Comparison between experimentally derived (blue) and simulated (red) 90–10% impulse response fall times for Lumogen Red 305-doped LSC (0.67 × 0.67 × 2cm) for a range of concentrations (*a* = 0.06–1.8cm^−1^). **g** Comparison between experimentally derived (blue) and simulated (red) 90–10% impulse response fall times for Lumogen Red 305-doped LSCs (*a* = 0.06cm^−1^) for a range of device lengths (*L* = 1–5cm)

To validate the time-domain MC algorithm, a set of carefully designed experiments were conducted covering a broad range of combinations within the device parameter space. Eleven devices were fabricated in total, the details of which are summarised in Table 1. The devices incorporated a range of fluorescence lifetimes and quantum yields, absorption and emission spectral overlaps, concentrations (quantified by absorption coefficient, *a*), and LSC lengths. Measurements and descriptions of fluorophore spectra, quantum yields, concentrations, and fluorescence lifetimes can be found in the Supplementary Material and Figs. S1, S2, and S3, respectively. Here, the measure of concentration is given in absorption coefficients, *a* (absorbance per unit length), for which the quoted values apply at 405 nm, the wavelength of the excitation beam used in our experiments. The LSCs consisted of poly(lauryl methacrylate) and ethylene glycol dimethacrylate co-polymer (PLMA-co-EGDM) host matrices doped with either Lumogen Red 305 (“slow”, single-exponential decay, *τ* = 6.5 ns, QY ≈ 93%) or caesium–lead–bromide-based perovskite nanocrystals (NCs; CsPbBr_3_—“fast”, multiexponential decay, *I* = *I*_0_*e*^−1^ in 3.8 ns, QY ≈ 65%), as pictured in Fig. 2a. The fluorophore concentration was adjusted to allow examination of a range of concentrations varying from tenuous (*a* = 0.06 cm^−1^) to optically dense media (*a* = 1.8 cm^−1^). All LSCs had a cross-section of 0.67 × 0.67 cm^2^, and their length *L* varied fivefold between 1 and 5 cm. Recipes for fabricating these devices can be found in the “Methods” section.

**Table 1:** Range of samples for which impulse responses were characterised and simulated

Flurophore	QY (Φ)	Lifetime (*τ*)	Absorption coefficient (*a*)	Length
Lumogen Red 305	0.93	Single exponential, 6.5 ns	0.06–1.8 cm^−1^	2 cm
Lumogen Red 305	0.93	Single exponential, 6.5 ns	0.06 cm^−1^	1–5 cm
CsPbBr_3_	0.65	Multiple exponential, *I* = *I*_0_/*e* in 3.8 ns	0.3 cm^−1^	2 cm

The experimental setup used to measure the impulse response of our system is schematized in Fig. 2b. The devices were excited at one end with a blue laser (Thorlabs NPL41B, 405 nm) modulated by a rectangular pulse of 37 ns width and a repetition rate of 5 MHz. This laser was chosen because its emission wavelength fell within the absorption spectra of both fluorophores used. The pulse width and repetition rate were chosen to allow the LSCs to switch fully on and off without interference between consecutive pulses. The excitation beam was parallel to the plane of the photodetector and orthogonal to the longer axis of the LSC. This minimised the chance of the photodiode not being illuminated directly, but instead only receiving light created by fluorescent emission events within the LSC.

A reference measurement of the square wave input pulse, directly illuminating the photodiode used in the experiments, is depicted in Fig. 2c. On the other hand, the insets in Fig. 2d, e show exemplar measurements through two characteristic LSCs, one with a fast, multiexponential response (CsPbBr_3_) and another with a relatively slow, single-exponential response (Lumogen Red 305). As expected, devices with slower responses result in more smoothing of the input pulse, while devices with a faster impulse response retain more high-frequency features from the input pulse, and display sharper rise and fall times. By calculating the deconvolution of the pulse after passing through an LSC with the directly illuminated pulse, the impulse response of the LSC can be derived. The impulse responses for both devices are shown in Fig. 2d, e, and indicate good agreement between the experiments and MC simulations. From these responses, we can extract important properties, such as the 90–10% fall time, which in turn can be used to predict the BW limits of such devices in communications systems, as discussed later in this paper.

Further comparison between the experimentally derived impulse responses with those obtained by the MC algorithm was performed for all devices in Table 1. Figure 2f compares the 90–10% fall times for LSCs of fixed length (*L* = 2 cm), but varying fluorophore concentration (*a* = 0.06–1.8 cm^−1^). Excellent agreement between experimental and simulated data can be observed, with the data matching consistently within 5%. Similarly, fixing the concentration of the devices (*a* = 0.06 cm^−1^) and varying the length from 1 to 5 cm again results in excellent agreement within 5% between the experiments and simulations; see Fig. 2f.

### Analytical solution for LSC impulse response

We are now ready to proceed to the analytical solution for the impulse response, which provides further insight into the interplay between the various mechanisms in dynamic LSC systems. To derive this solution, two assumptions are made: (i) the pdf of fluorescence events follows single-component exponential decay (a requirement that is relaxed in the Supplementary Material, on the section on bandwidth limits for multiexponential decay, where equations are generalised for multiexponential distributions), consistent with Eq. (2), and (ii) time delays due to photon propagation can be neglected; thus, all contributions in the time domain emanate from fluorescent decays. We make the latter approximation as the longest path a ray can travel along a device during total internal reflection at the critical angle. At this angle, the optical path from one end of the LSC to the other is approximately *nL*, where *n* is the refractive index of the host matrix. As such, in order for 1 ns to contribute to the time delay for a typical host matrix with *n* = 1.4, *L* must exceed 15 cm, which is much longer than the dimension of the tested devices. The latter condition refers to an optimum scenario and sets a lower limit for the time delay that occurs in dynamic LSCs in that if its additional contribution were to be accounted for, it would serve only to broaden the system’s impulse response.

The assortment of photons that reach the edge of an LSC can be grouped according to the number of fluorescence events that each photon undergoes. In sufficiently tenuous media, a single absorption event occurs, so the probability, *P*_1_(*t*), of photons reaching the desired edge of the LSC follows an exponential pdf, i.e., *P*_1_(*t*) = 1/*τ* *e*^(−*t*/*τ*)^, as a direct consequence of Eq. (2), where the coefficient 1/*τ* normalises the pdf to unity area. However, in denser media, a fraction of these photons undergo multiple absorption events before reaching the edge. As the probability of a decay occurring between *t* and *t* + *δt* is statistically independent of any previous fluorescence events, the cumulative pdf for two events can be calculated by the convolution of the pdfs of each individual event^33^. For two emission events, this is given by *P*(*t*) = *P*_1_(*t*) ⊗ *P*_2_(*t*) = 1/*τ* *e*^−t/τ^ ⊗ 1/*τ* *e*^−*t*/*τ*^ = 1/*τ*^2^ *te*^−*t*/*τ*^. By mathematical induction, the general form of the total weighted pdf is easily shown to correspond to a polynomial series multiplied by the same exponential term, as shown in Eq. (5).

5 $$P_{\mathrm{{weighted}}}\left( t \right) = \mathop {\sum }\limits_l \frac{{A_lt^{\left( {l - 1} \right)}}}{{{\Gamma}\left( l \right)\tau ^l}}e^{ - \frac{t}{\tau }}$$

where Γ(*l*) = (*l* − 1)! for integer values of *l*. Here, *l* counts the number of absorption events undergone by photons received at the collection face of the LSC. Each term has been multiplied by weights *A*_*l*_, coefficients representing the proportion of incident photons that are successfully collected at the photodiode edge and have undergone *l* absorption events (it is reminded that since the source and photodiode are in an orthogonal arrangement, no direct photons from the source arrive at the photodiode, and so the summation starts from *l* = 1). This normalisation makes the total probability equal to the sum of all coefficients *A*_*l*_. In this sense, the sum of all *A*_*l*_ is equal to the external optical efficiency (ratio of successfully concentrated photons to total incident photons^25^ of the device). Hence, Σ*A*_*l*_ ≤ 1, the inequality expressing the physical loss of photons due to the fluorophore non-unity quantum yield and via escape cone losses within the device.

**Fig. 3:**
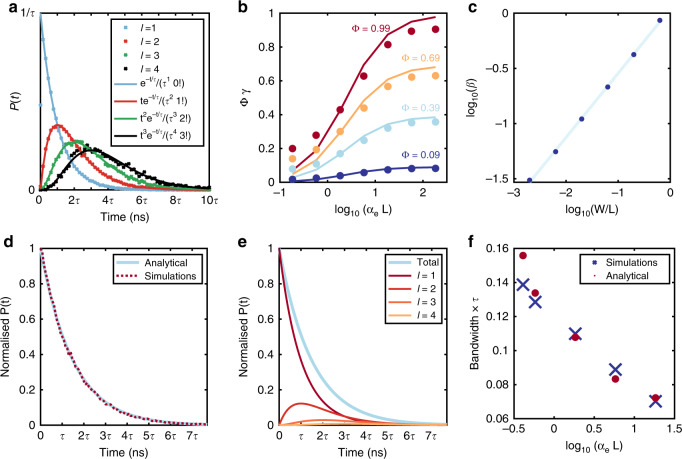
Analytical solution to impulse response prediction. **a** Comparison between normalised time curve contributions from simulations (solid line) grouped by the number of fluorescence events, *l*, compared to the analytical solution of probability density functions for *l* = 1 (blue), 2 (red), 3 (green), and 4 (black) as a function of time in multiples of *τ*. **b** Simulated (points) and fitted (solid lines) for Φ*γ* plotted against the logarithm of *α*_*e*_*L* for a range of quantum yields: (from top to bottom) QY, Φ = 0.99, 0.69, 0.39, and 0.09. **c** Dependence of fitting constant *β* as a function of the ratio of device width to length. **d** Comparison between simulated (dashed) and analytical (solid) solutions for the impulse response of an example LSC as a function of time. **e** Deconstruction of the analytical impulse response from **d** into the contributions from photons that have undergone *l* absorption events for *l* = 1, 2, 3, and 4. **f** Comparison of normalised bandwidths for simulated (blue crosses) and analytically predicted (red circles) for a series of LSCs with varied fluorophore concentrations

We further confirm that the time profile follows this pattern by splitting the output of the MC model for an LSC into its elementary contributions from each reabsorption event and comparing this model to the analytical solution. As Fig. 3a shows, the contributions (normalised to unity area) derived from the MC model for *l* = 1–4 (symbols) precisely follow the predicted patterns of *t*^(*l* − 1)^ *e*^(−*t*/*τ*)^/(*l* − 1)!*τ*^*l*^ (solid lines). Note that these are universal results, independent of the fluorescence lifetime, and so are presented in multiple intervals of *τ*. It becomes evident from Fig. 3a then that every time a new reabsorption event occurs, the optical impulse response of the system is successively broadening. In the multiexponential case, the analysis above still remains valid, but Eq. (5) has to be replaced by the successive application of the convolution function of a multiexponential pdf, which can easily be performed semi-analytically. Regardless, the salient characteristic of impulse response broadening with the addition of extra reabsorption events is still true. The limiting case for which only one absorption event happens defines, therefore, the maximum device BW. Moreover, for (single) exponential time responses, the bandwidth of a device (in GHz) can be approximated by 0.35 divided by the 90–10% drop of the impulse response (in ns)^34,35^. As such, the fundamental upper BW limit for arbitrary LSC devices can be approximated by Eq. (6), which can be further simplified to Eq. (1).

6 $${\mathrm{BW}}\left( {\mathrm{{GHz}}} \right) \le \frac{{0.35}}{{\tau \left( {\ln \left( {0.9} \right) - \ln \left( {0.1} \right)} \right)}}\left( {\mathrm{{ns}}} \right)$$

An alternative route to derive the same result is by Fourier transforming the system’s impulse response and identifying the −3 dB frequency (*ω*_3 dB_), as presented in the Supplementary Material (“Bandwidth limits for multiexponential decay”). This is particularly useful for multiexponential decay systems for which *τ* in Eq. (1) can now be substituted by the effective time constant 〈*τ*〉 = 1/*ω*_3 dB_. Equation (6) is in agreement with the bandwidth for an RC-limited photodiode^36^, represented by the circuit diagram shown in Fig. 1b, with the RC constant being replaced by the fluorescence lifetime *τ* (or 〈*τ*〉 for multiexponential decays). MC simulations for a range of devices providing further evidence that these limits hold true are presented in the Supplementary Material and Fig. S4a for single-exponential fluorophores and Fig. S4b for bi-exponential fluorophores.

Further completing the derivation for the BW limits, we semi-analytically define the coefficients *A*_*l*_ and hence resolve the contribution from each reabsorption event to the system’s impulse response. Starting from *A*_1_, this is given explicitly by Eq. (7) (ref. ^37^).

7 $$A_1 = \left( {1 - R} \right)\left( {1 - e^{ - tc{\it{\epsilon }}}} \right){\Phi}\left( {1 - \gamma } \right)\eta$$

Here, (1 − *R*)(1 − *e*^−*tcϵ*^) corresponds to the portion of the incident light that is transmitted and absorbed within the LSC. This quantity is calculated by using Beer–Lambert’s law, where *R* is the host matrix reflection, *t* is the device thickness, *c* is the fluorophore concentration in mol dm^−3^, and *ϵ* is the molar attenuation coefficient. In addition, Φ is the quantum yield of the fluorescent material, *γ* is the proportion of light re-absorbed in the LSC, and *η* is the waveguiding efficiency, i.e., the portion of photons reaching the LSC edge (the rest being lost due to escape cone losses). For light incident normally to the LSC, *R* = ((*n* − 1)/(*n* + 1))^2^. The waveguiding efficiency to a single face of an LSC, *η*, has been approximated in previous literature^38^ and is given in Eq. (8).

8 $$\eta = \frac{1}{2}\left( {1 - \sqrt {1 - \frac{1}{{n^2}}} } \right)$$

While this is an approximation and may not be accurate in all cases, it gives a reasonable estimate for our calculation of *A*_1_. In practice, *A*_1_ is useful for the prediction of collection power, but not the bandwidth. The time-domain response is affected not by *A*_1_, but by the relative values of *A*_1_ to another *A*_*i*_.

For *l* > 1, it is straightforward to calculate the rest of the coefficients *A*_*l*_, as the system obeys Markov chain statistics, whereby its (*l* + 1)th state probability depends solely on the *l*th state and is independent of any previous history. In addition, every time a photon is re-emitted, it has the same probability, *η*, to be guided to the edge of the LSC. These conditions dictate a recursive relation between *A*_(*l* + 1)_ and *A*_*l*_, with the two connected simply by the joint probability of a photon be re-absorbed and then emitted, as shown in Eq. (9).

9 $$\frac{{A_{l + 1}}}{{A_l}} = \gamma {\Phi}$$

MC simulations confirming that this relation is constant for all *l* > 1 are presented in Supplementary Material and Fig. S5.

It has been shown in the literature^37^ that the quantity (1 − *γ*) is related to the length of the device, *L*, and a weighted average absorption cross-section of the emission spectrum of the luminophore, *α*_*e*_, via Eq. (10).

10 $$\left( {1 - \gamma } \right) = \frac{1}{{1 + \beta \alpha _eL}}$$

with,

11 $$\alpha _e = \frac{{{\smallint }S_{\mathrm{{PL}}}(\lambda )\alpha (\lambda ){\mathrm{d}}\lambda }}{{{\smallint }S_{\mathrm{{PL}}}(\lambda ){\mathrm{d}}\lambda }}$$

Here, *β* is an empirical constant that relates to the specific LSC geometry, and *α*(*λ*) = (1 − *e*^−*tcϵ*(*λ*)^). To validate this, we simulated a series of LSC devices with a range of lengths and quantum yields, but fixed square cross-sections. From our simulations, we extracted the portion of emitted light that is absorbed and re-emitted as a function of *α*_*e*_*L*. Here, *α*_*e*_ is used as the measure for concentration in order to give a generalised non-fluorophore specific quantity for concentration and overlap between absorption and emission spectra. We then fitted the simulated *γ*Φ to Φ(1 − (1 + *βα*_*e*_*L*)^−1^), fitting for *β*. We found that the best fit for *β* is consistent for all quantum yields, giving values of *β* in the range 0.405 ± 0.05 for a fixed ratio of device length to width (and a square cross-section). As shown in Fig. 3b, the fitted solution fits the simulated ratio between *A*_*l*_ and *A*_(*l* + 1)_ well, especially at high concentrations, where the reabsorption is high and where the contributions to multiple absorption events thus become significant.

To take into account the geometrical effects of *β*, we explored the dependence on the ratio of device width to length, discovering a logarithmic dependence, as shown in Fig. 3c. We fitted the value of *β* relative to *W*/*L* by Eq. (12) with values of constants *A* = 0.581 and *B* = 0.036, calculated by taking the gradient and intercept of the best fit line for the values of *β* shown in Fig. 3c. The *R*^2^ for this fit was found to be 0.97.

12 $$\beta = 10^{A{\mathrm{log}}_{10}(W/L) + B}$$

Having established approximations for *A*_1_ and the ratio between *A*_l_ and *A*_(*l* + 1)_, we have constructed a full semi-analytical solution for the amount and arrival time of photons reaching the LSC edge. Note that Eqs. (8)–(11) are independent of the fluorescence characteristics and thus applicable for fluorophores of any decay characteristics. Next, we compare the semi-analytical solution to MC simulations. In Fig. 3d, we show a comparison of the impulse response curves for an example LSC (Φ = 0.75, *β* = 0.405, *α*_*e*_ = 0.37, *L* = 5 cm), as predicted by our semi-analytical solution (solid blue line) and the MC simulation (dashed red line), with the two being in excellent agreement. For reference, we show in Fig. 3e the semi-analytical solution for the same LSC, broken down into the contributions from photon groups that have undergone *l* absorption events. As evident from the difference between the total impulse response (blue) and the contribution from single absorption events (dark red), reabsorption may have a detrimental impact on the system’s impulse response in denser media and needs to be carefully accounted for. To make this point clearer, we show in Fig. 3f a comparison between simulated and semi-analytically predicted normalised bandwidths (BW × *τ*) for a set of LSCs with varied concentrations. As expected, the bandwidth decreases as the concentration is increased. The same conclusion is reached for LSCs whose concentration is kept constant but whose Stokes shift changes, as shown in the Supplementary Material, Fig. S7. As expected, the performance of the devices improves (bandwidth increase) with increasing Stokes shift due to decreasing overlap between absorption and emission. Notably, the predictions from the semi-analytical solution in Fig. 3f match within 5% of the simulated bandwidths at all concentrations apart from the lowest (where the error is 12%). At these concentrations, the reabsorption is low, and the use of the simpler Eq. (6) is more appropriate.

### Analytical solution for the LSC impulse response

Having both simulated and measured the impulse response, we can relate this quantity to communication system parameters, demonstrating the use of LSCs in a proof of concept communications system. We did this by setting up an experiment in which a 520 nm LED is modulated to transmit data, driven by a function generator (Tektronix AFG3022B) that outputs a previously generated pseudo-random binary sequence. The LSC samples were used to collect the light emitted by the LED and concentrate it into an attached photodiode, the output of which we measured on an oscilloscope that was used to digitise the data. Further details of the experiment and equipment used can be found in the “Methods” section.

For these experiments, we used three Lumogen Red 305-based samples, for which the measured impulse response fall times, *T*, were 17.5, 23.5, and 22.5 ns for samples A, B, and C, respectively. As discussed, the bandwidth, B, of such a device can be approximated by the equation BW (GHz) = 0.35/*T* (ns), and the simulated impulse response times correspond to predicted bandwidths of 20, ≈15, and 15.5 MHz.

**Fig. 4:**
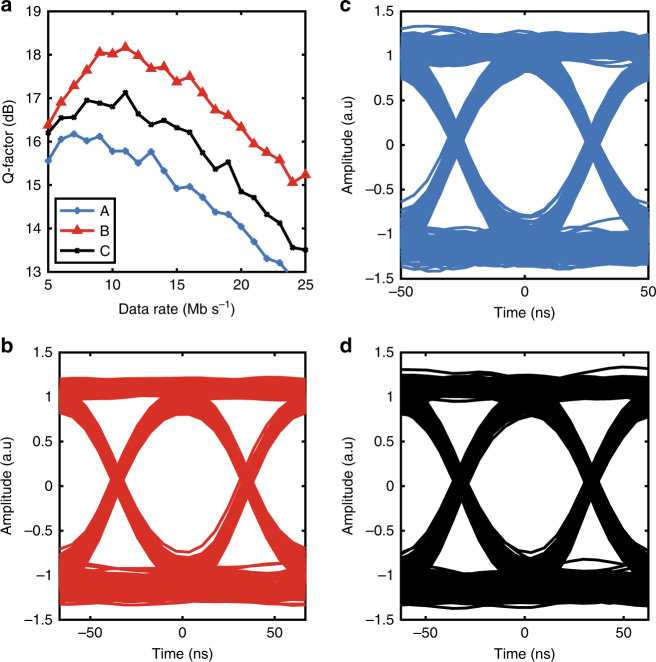
Communication system demonstration. **a** The measured *Q*-factor of the link as a function of data rate for the three samples tested. Each of the *Q*-factors shows a similar profile: an initial increase due to the penalty caused by the coupling capacitors at the transmitter and receiver^46^ followed by a peak, and then consistent degradation due to the low level of light recovered from the devices and its ratio to the noise equivalent power of the photodetector. Nevertheless, to show the performance of the devices under test, we illustrate the eye diagrams for **b** sample A at 20 Mb/s, **c** sample B at 15 Mb/s, and **d** sample C at 16Mb/s. Each of these clearly shows a clear eye opening at the centre point of the symbol period and a high degree of symmetry within both rise and fall times

No bit errors were observed in the system; therefore, we examined the *Q*-factor as the key performance metric, as it is closely related to the statistical bit-error rate and SNR^39^. The *Q*-factor is given by *Q* = (*μ*_1_ − *μ*_0_)*/*(*σ*_1_ + *σ*_0_)^−1^, where *μ*_*x*_ and *σ*_*x*_ are the mean and standard deviations of the two logical signal levels, respectively. The *Q*-factor results are illustrated in Fig. 4a and clearly show that the performance of each of the LSCs effectively demonstrates that the data can be recovered successfully on each link. As expected, the *Q*-factor starts to degrade as the data rate increases due to the low amount of optical power recovered from the LSCs (sample A: 12.6 μW, sample B: 30 μW, and sample C: 27 μW) and the increasing signal (plus noise) bandwidth means increased signal power must be obtained to maintain equivalent performance. Interestingly, sample A, which has the highest bandwidth, does not exhibit a superior *Q*-factor gradient to that of the other samples, which may be expected. The reason for this is that sample A also has the lowest optical power output, and this dominates the performance of the sample due to operation close to the noise floor of the test setup.

To confirm that the rise times of the system correspond to the values mentioned above, the eye diagrams of the received signals are illustrated in Fig. 4b–d for samples (b) A, (c) B, and (d) C. The data rates of the three eye diagrams were set at the bandwidths predicted by the time-domain response outlined above, i.e., sample A: 20 Mb/s, sample B: 15 Mb/s, and sample C: 16 Mb/s. Setting the rate equivalent to the bandwidth gives rise to a clean eye opening in the centre of the symbol period plus a highly symmetric rise and fall time. This indicates that the communications link is free from asymmetric interference and closely follows the shape expected for a Nyquist-I low-pass response when the bit rate is set equal to the bandwidth^40^.

## Discussion

**Fig. 5:**
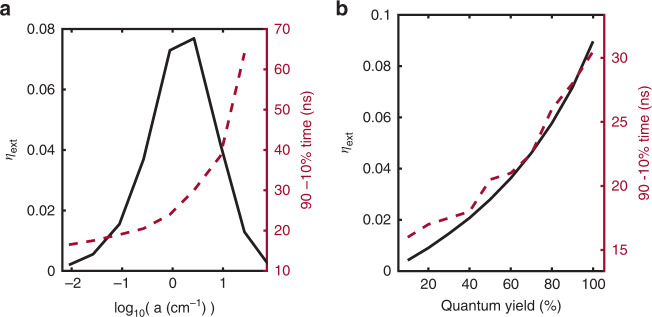
Effects of luminescent solar concentrator parameters on impulse response. **a** (Black solid line) simulated external optical efficiency, *η*_ext_, as a function of the logarithm of the absorption coefficient of the devices for fixed device parameters. (Red dashed line) simulated fall time of the impulse response of devices as a function of the logarithm of the absorption coefficient. **b** (Black solid line) simulated external optical efficiency, *η*_ext_, as a function of quantum yield for a Lumogen Red 305-doped device with fixed concentration and dimensions. (Red dashed line) simulated fall time of the impulse response as a function of quantum yield

The time-domain platform described in this paper can be used to arrive at several important conclusions, which are not necessarily aligned with the usual optimisations used in the LSC field. For example, in the case of maximising output LSC power, there is a series of guidelines that are generally consistent throughout the field; the fluorophore quantum yield should be as close to unity as possible, and the concentration of fluorophore should be high enough such that a large amount of light is absorbed, yet not so high such that the effects of reabsorption losses start to overtake the benefits of the additional absorbed light. However, these optimisations do not result in the sharpest possible impulse responses and, in turn, the largest possible bandwidths. For example, when modelling a 0.67 × 0.67 × 5 cm Lumogen Red 305-doped device, we can see that, as expected, the external optical efficiency *η*_ext_ (defined in the “Methods” section) increases, peaks, and then decreases as reabsorption begins to dominate, as shown in Fig. 5a. The 90–10% fall time of the impulse response, however, does not follow the same trend. As seen in Fig. 5a, the fall time continually increases with concentration. This result is intuitive, as the probability of multiple absorption events is increased with an increase in concentration. However, it does provoke additional considerations when incorporating LSCs for use in communications systems. The result implies an interplay between the optimisation of the output power and time response. In a similar manner, the response of the devices in the time domain is not necessarily improved by an increase in quantum yield. We simulated the relationship between both the external optical efficiency and the impulse response to the quantum yield, fixing all other parameters, as shown in Fig. 5b. As expected, the external optical efficiency increases with an increase in quantum yield; however, less intuitively, the impulse response becomes slower as the quantum yield increases. Longer impulse responses are associated with a larger contribution of photons that undergo multiple absorption events. In the case of lower quantum yields, any photons that undergo multiple absorption events are less likely to then be re-emitted; correspondingly, there is a greater proportion of events undergoing just one absorption event, giving a faster impulse response. Once again, there is an interplay between output power and bandwidth that should be considered.

Notably, there are further links between the fluorophore emission lifetime and quantum yield. Typically, for comparable fluorophores, a longer fluorescence lifetime corresponds to higher quantum yields due to the reduction of non-radiative decay paths^24^. For example, fluorophores that have been subjected to UV degradation exhibit lower quantum yields and shorter fluorescence lifetimes due to an increase in non-radiative decay.

As discussed, these parameters affect the SNR and bandwidth of the system and, as such, should be carefully considered and tailored according to the specific application. These trade-offs highlight the importance of modelling the dynamic performance of LSC devices when designing LSC-VLC systems.

To conclude, in this paper, we have demonstrated two experimentally verified tools for the analysis of LSCs in the time domain: an analytical solution and a MC method algorithm. By means of the analytical solution, we have outlined the limits of LSC bandwidth and provided a simple method for the calculation of the LSC impulse response for basic LSC configurations. To complement this, the MC methods algorithm provides a solution for more complex systems, such as large devices, complicated geometries or compound systems, paving the way for the development of novel, efficient, and fast free-space optical communication systems.

## Methods

### Monte Carlo methods platform

Our MC ray tracing platform has been used to accurately predict the efficiency and light propagation through LSCs in our previous publications^28^. Light propagation is modelled on a photon-by-photon basis, with potential reflection or transmission events at interfaces or fluorescence absorption/emission events throughout the medium. The probabilities of reflection or transmission are calculated according to Fresnel’s laws. The distance to each absorption event is given by an inverse sampling method where the distance is based on a random number generator and Beer–Lambert law. Extinction coefficients for each luminophore are calculated based on measurements from a UV–Vis photospectrometer (Shimadzu UV-1800). The emission spectra of the luminophores are obtained from measurements using TCSPC spectroscopy (LifeSpec-ps, Edinburgh Instruments). The probability of re-emission, which is the quantum yield, is calculated based on LSC efficiency measurements and normalised to take into account device dimensions following the methods described in our previous publication^25^. The number of photons simulated per device ranges between 0.1 and 10 million according to the required resolution. In the case of low efficiency LSCs, a smaller proportion of photons reaches the edges, so a higher number of photons needs to be simulated to achieve convergence with equivalent degrees of accuracy.

### Caesium-oleate synthesis

Caesium carbonate (Cs_2_CO_3_, 0.204 g, Aldrich) was loaded into a 100 mL three-neck flask along with octadecene (ODE, 10 mL, Aldrich) and oleic acid (OA, 0.625 mL, Aldrich). This was then dried for 1 h at 120 °C and then heated under nitrogen to 150 °C until all Cs_2_CO_3_ reacted with OA. The resultant Cs-oleate was clear and was stored at 100 °C under nitrogen before injection.

### Synthesis of CsPbBr_3_ nanocrystals

ODE (5 mL) and lead bromide (PbBr_2_ 0.069 g, TCI) were loaded into a 25 mL three-neck flask and dried under vacuum for 1 h at 120 °C. Oleylamine (0.5 mL, Aldrich) and OA (0.5 mL, Aldrich) were injected at 120 °C under nitrogen. From this point, the solution was always kept under nitrogen. After complete solubilisation of PbBr_2_, the temperature of the solution was raised to 140 °C, and the Cs-oleate solution (0.4 mL, 0.125 M in ODE, prepared as described above) was quickly injected. The colour of the solution rapidly turned bright green, and NCs were allowed to grow for 5 s before the reaction was quenched in an ice-water bath. The NCs were then separated from the reaction solution and purified via centrifugation at 6000 RPM for 5 min. Subsequently, the supernatant was discarded, and 5 mL of toluene was added to the precipitates to disperse the NCs. The solution of NCs was then stored in a refrigerator for 24 h. A final centrifugation step was then used to remove the precipitate before it was used for the experiments. The synthesis of Cs-oleate and NCs follows previously published recipes^23,41^.

### Fabrication of luminophore-doped LSCs

PLMA-co-EGDM-based LSCs were fabricated following synthesis techniques described in previous publications^42,43^. A solution comprising a 5:1 (by weight) ratio of LMA (containing 500 p.p.m. MEHQ as an inhibitor, 96%, Sigma-Aldrich) and EGDM (98%, containing 90–110 p.p.m. monomethyl ether hydroquinone as an inhibitor, Sigma-Aldrich) was added to UV protective glass vials, mixed, and sonicated for 10 min in a chilled ultrasonic bath. A 1% (by weight) quantity of UV initiator, (diphenyl(2,4,6-trimethylbenzoyl)phosphine oxide (97%, Sigma-Aldrich) and the desired concentration of luminophore (Lumogen Red 305 (BASF) or CsPbBr_3_ NCs) were added, and the solution was further mixed and sonicated for 20 min. The solution was then poured into a mould consisting of two glass sheets separated by a silicone spacer. The mould was illuminated under ultraviolet light for 15 min before being left in the dark for 45 min to reduce cracking. The glass sheets were then manually removed, and the LSCs were cut to size using a laser cutter.

### Impulse response measurements

For measurements of the impulse response of the LSC devices, we illuminated the LSC with a pulsed nanosecond laser diode (Thorlabs NPL41B) perpendicular to the long axis of the device. At the opposite end, we positioned the LSCs such that the light escaping the end was coupled into a photodiode (Thorlabs PDA10A-2). The photodiode output was digitised with an oscilloscope (Agilent Infinium, 54830B). The pulse, both directly illuminating the photodiode and at the output of the LSC, was sampled at 2 Gb/s and averaged 2048 times.

### Communication system measurements

To validate the model and the measured rise and fall times, it is necessary to validate the performance in terms of a communication system. Since the nanosecond laser diode used to obtain the rise time measurements cannot be modulated with an independent, random sequence of pulses, it was replaced with a 520 nm (green) LED (Vishay Semiconductors, VLMTG1300-GS08), referring to the experimental test setup in as reported in prior art^44^, but without the equaliser. We selected a diode with an emission wavelength of 520 nm, which falls close to the peak of the absorption band of Lumogen Red 305. The bandwidth of the LED was measured to be ≫50 MHz, as reported in the literature^45^, which is much larger than the bandwidth of the LSC devices used, so it did not act as a limit to the system. The LED was DC biased at 90 mA and driven with a 2 Vpp amplitude as in prior art^45^. The purpose of this work was not to extend the state-of-the-art data rate of a VLC system, but to examine the performance of the LSC as an element in the system. Therefore, the test was independent of the modulation format, so we chose binary pulse-amplitude modulation for simplicity, generated a $2^{10} - 1$ length sequence of pseudo-random data and varied the data rate from 5 to 25 Mb/s. The rate was limited by the function generator used to generate the pulsed data (Tektronix AFG3022B), which was frequency limited to a maximum of 25 MHz. The photodiode (Thorlabs PDA10A-2) used to absorb the light collected by the LSC has a bandwidth of 150 MHz, and the oscilloscope used to digitise the data was an Agilent Infinium 5VLC4830B with 600 MHz bandwidth. The sampling frequency was set to 2 GS/s, providing a significant oversampling rate, and at least $10^6$ bits were captured, consistent with other work in the literature^44^.

## Supplementary information

Supplementary material
