# Supplementary material for: Bandwidth limits of luminescent solar concentrators as detectors in free-space optical communication systems
Source: Light Sci Appl. 2021 Jan 1;10:3. doi: 10.1038/s41377-020-00444-y (PMC7775919; doi:10.1038/s41377-020-00444-y)
Supplement: Supplementary file 1 — Supplementary material [file 41377_2020_444_MOESM1_ESM.pdf]

Supplementary materials

Mark Portnoi[1], Paul Anthony Haigh[2], Thomas J Macdonald[3,4], Filip Ambroz[4], Ivan P Parkin[4], Izzat Darwazeh[5], and Ioannis Papakonstantinou[1,*]

[1]Photonic Innovations Lab, Department of Electronic and Electrical Engineering, University College London, London, WC1E 7JE, UK
[2]School of Engineering, University of Newcastle, Newcastle, NE1 7RU, UK
[3]Department of Chemistry, Imperial College London, London, W12 0BZ, UK
[4]Department of Chemistry, University College London, London, WC1H 0AJ, UK
[5]Department of Electronic and Electrical Engineering, University College London, London, WC1E 7JE, UK
[*]Corresponding author: i.papakonstantinou@ucl.ac.uk

Characterisation of Fluorophores and LSC devices

Absorption and Emission Spectra

The absorption coefficient, a, of a material is defined as a measure of the absorbance per unit length, and is proportional to fluorophore concentration according to Beer-Lambert law. Absorption Coefficients for each fluorophore and LSC were measured using a UV-Vis spectrophotometer (Shimadzu UV-1800) using an undoped polymer sample of the same thickness as reference to remove the effects of reflections/absorption off the host matrix. Emission spectra were measured using with low concentration samples using time correlated single photon count spectroscopy (TCSPC) (LifeSpec-ps, Edinburgh Instruments) to reduce the effects of spectral shift due to reabsorption. Figures S1a and S1b show the absorption coefficients measured for the Perovskite and Lumogen Red 305 devices discussed in the main text. Here the Perovskite based LSC is the only $CsPbBr_3$ LSC discussed ($2 \times 2 \times 0.67$ cm), whilst the values for the Lumogen Red 305 based LSC is the lowest concentration device of the series of 5 devices where concentration is varied ($2 \times 2 \times 0.67$ cm). Absorption coefficients (absorbance per unit length which depends on fluorophore concentration) for the other Lumogen devices can be calculated by multiplying throughout by the ratios of listed absorption coefficients at 405 nm.

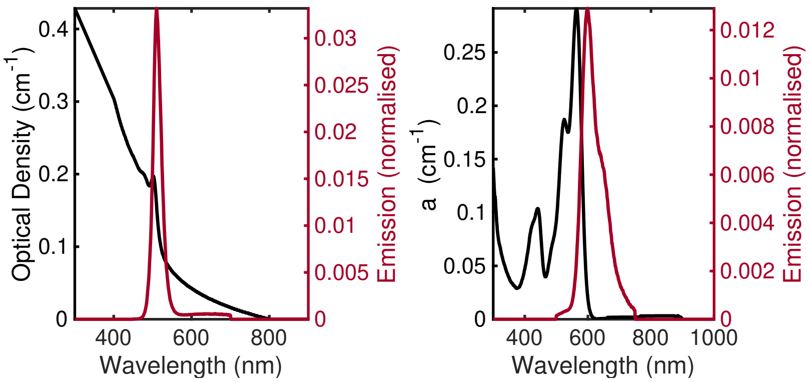

(a) (b)

Figure S1. Measured absorption coefficients as a function of wavelength (black) and normalised emission spectra as a function of wavelength (red) for (a) the $CsPbBr_3$ quantum dot LSC discussed in the main text and (b) a Lumogen Red 305 based LSC. Here the absorption coefficient is shown for the lowest concentration Lumogen Red 305 device discussed in the main text.

Fluorescence lifetimes

Fluorescence lifetimes were measured using time correlated single photon count spectroscopy (TCSPC). In the case of the fluorescence decay curve of Lumogen Red 305, this was found to be a single component exponential, which can be seen by plotting the decay on a logarithmic scale, as shown in Figure S2a. The fluorescence lifetime can be calculated by fitting a

straight line to the decay curve and taking the gradient. The gradient, m, is related to the lifetime, τ by relation $m = -1/\tau$. For this particular measurement of Lumogen Red 305 doped PLMA-co-EGDM the lifetime was found to be 6.5 ns.

In the case of the $CsPbBr_3$ perovskite quantum dots, the decay does not follow a simple single exponential, as when plotted on a logarithmic scale the decay is not linear, shown in Figure S2b. As such we fit a multiple component exponential decay with 2 exponential components, yielding the equation $I(t) = a_1 exp(-t/\tau_1) + a_2 exp(-t/\tau 2)$. As shown in Figure S2c, we achieve an excellent fit ($R^2 > 0.99$) with values of a_1, τ_1, a_2 and τ_2 found to be 0.1735, 16.21 ns, 0.8215 and 2.977 ns respectively. The time for intensity to drop for this 1/e is approximately 3.8 ns, significantly faster than the Lumogen Red 305 fluorophores. As discussed in the main text, we sample this multiple exponential decay function when simulating the time-curve of a single fluorescence event.

The measured fluorescence lifetimes may appear slightly faster than the true value due to an element of self absorption within the measured samples, however the samples used in these measurements were low concentration and small offcuts in size to reduce these effects as much as possible.

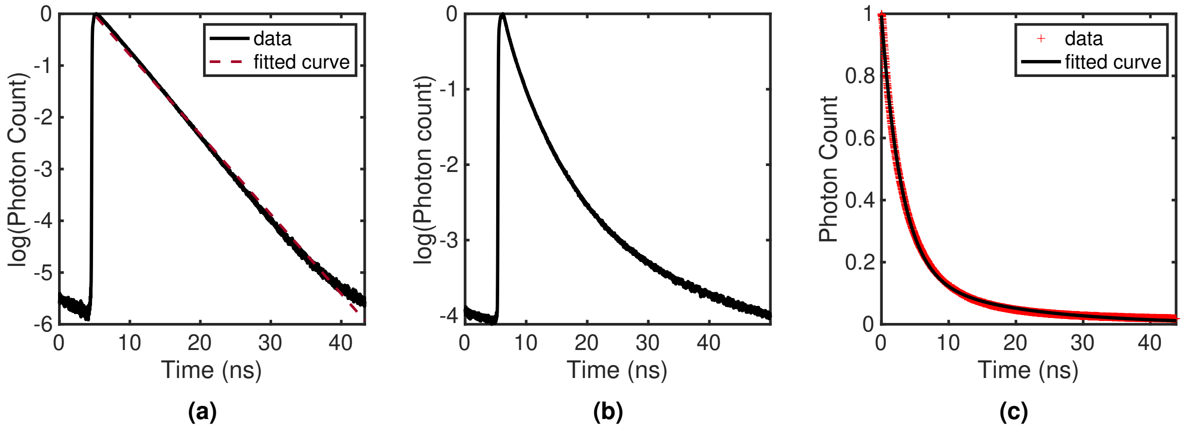

Figure S2. (a) Normalised TCSPC measured decay curve for Lumogen Red 305 doped PLMA-co-EGDM LSC plotted on a logarithmic scale, and fitting (dashed line). (b) Normalised TCSPC measured decay curve for $CsPbBr_3$ doped PLMA-co-EGDM LSC plotted on a logarithmic scale. (c) Normalised TCSPC decay curve for $CsPbBr_3$ quantum dots (black), and fitting to a two component multi-exponential decay curve.

Quantum Yield Measurements

In order to establish the quantum yields of the two fluorophores when encapsulated in PLMA-co-EGDM we measured the losses and efficiency of both Lumogen Red 305 and $CsPbBr_3$ doped devices using a monochromatic light source, integrating sphere and photo-spectrometer, following the methods previously used and experimentally verified in our previous publication[1]. The method measures total quantum yield losses and does not distinguish between losses from a single and multiple absorption events, and also is not normalised to the geometry of the LSC devices. However by using the Monte-Carlo methods model we can take these factors into account, generating the a quantum yield that results in corresponding losses and internal efficiency as close as possible to the measured values, given fixed physical dimensions and absorption coefficients.

Figures S3a and S3b show the comparisons between measured and simulated photon fates for the (a) Lumogen Red 305 and (b) $CsPbBr_3$ devices respectively. The possible photon fates are (blue) Internal Optical Efficiency, η_{int}, defined as the ratio of the number of successfully waveguided photons to the number of absorbed photons, (red) quantum yield losses, i.e. losses of photons as heat due to the non-unity quantum yield of the fluorophore, and (yellow) Escape cone losses, i.e. photons that escape the larger faces of the LSC.

In this case the $CsPbBr_3$ doped device was a $3 \times 3 \times 0.67$ cm with absorption coefficient at 405 nm of 0.29 cm^{-1}, resulting in a calculated quantum yield of 0.65. The Lumogen Red 305 doped device $4 \times 4 \times 0.67$ cm with absorption coefficient at 405 nm of 0.09 cm^{-1} resulting in a calculated quantum yield of 0.93.

Bandwidth Limits for Multiexponential Decay

Suppose we are dealing with fluorophores exhibiting multiexponential decay characteristics given by:

$$I(t) = \sum_i a_i e^{-t/\tau_i}, \qquad (1)$$

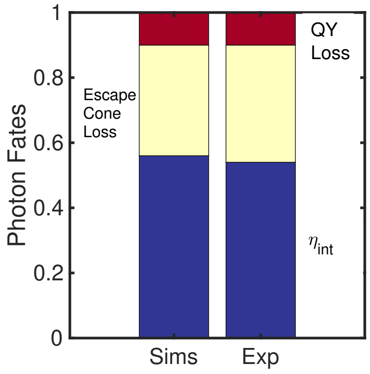

(a)

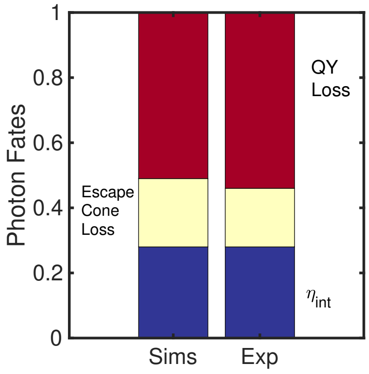

(b)

Figure S3. Simulated (left) and Experimentally measured (right) photon fates for (a) Lumogen Red 305 (b) $CsPbBr_3$ doped devices. Photon fates are split into Internal Optical Efficiency, η_{int} (blue), Escape Cone Losses (yellow), and Quantum Yield Losses (red).

This is conveniently normalised to unity amplitude at $t = 0$ implying $\sum_i a_i = 1$. As discussed in the main text, the BW limits are considered for devices for which re-absorption events are omitted. Fourier transforming Equation 1 gives

$$I(\omega) = \sum_i \frac{a_i \tau_i}{j\omega\tau_i + 1}. \tag{2}$$

In the above equation, the amplitude is normalised to unity for DC values, implying that $\sum_i a_i = 1$. The 3dB bandwidth is calculated at the angular frequency ω_{3db} for which the square of the amplitude drops to a half of its DC value and so, is given by

$$|I(\omega_{3db})|^2 = |\sum_i \frac{a_i \tau_i}{j\omega_{3db}\tau_i + 1}|^2 = \frac{|I(\omega = 0)|^2}{2}. \tag{3}$$

For a bi-exponential distribution, which is obeyed by a large number of fluorophores, this reduces to the simple quadratic equation

$$\frac{\omega_{3db}^2(\tau_1\tau_2)^2/(a_1\tau_1 + a_2\tau_2)^2 + 1}{(\omega_{3db}^2\tau_1^2 + 1)(\omega_{3db}^2\tau_2^2 + 1)} = \frac{1}{2}. \tag{4}$$

By definition[2], the effective lifetime is given by $\langle\tau\rangle = 1/\omega_{3db} = 1/2\pi B$. Inverting this equation, retrieves Equation 1 in the main text. As an example, in the $CsPbBr_3$ quantum dot case, we found earlier that $a_1 = 0.1735$, $\tau_1 = 16.21$ ns, $a_2 = 0.8215$ and $\tau_2 = 2.977$ ns. The effective lifetime is then calculated from Equation 4 to be $\langle\tau\rangle = 11.1$ ns and the corresponding bandwidth limit $B = 14.4$ MHz.

The analysis for the LSC impulse response presented in the main text is also valid. The only modification relates to Equation 5 for the calculation of the pdf convolution. However, this is a trivial step which can be performed in either the time or frequency domains (calculations not shown here).

Verification of Limits

In the main text we derive that the bandwidth, B, of LSCs is limited by the equation $B \leq 1/2\pi\tau$. In order to provide further evidence of these limits holding true we used the MC ray-tracing algorithm to simulate a series of 27 LSC devices of varied length ($L = 2cm, 20cm, 40cm$), concentration ($\alpha_e = 0.037cm^{-1}, 0.37cm^{-1}, 3.7cm^{-1}$) and lifetime ($\tau = 5ns, 10ns, 15ns$). Here all the devices have a cross-section of $1cm \times 1cm$ and a fluorescence quantum yield of 0.99. Also, α_e is a non-fluorophore specific measure of concentration and is defined in Equation (11) of the main text. Figure S4a shows the quantity $B \times \tau$ plotted against $log_{10}(\alpha_e L)$ for the aforementioned devices. It also shows the predicted limit for $B \times \tau = 0.1593$. As seen in the Figure, all simulated devices fall below this limit. As expected, devices with larger $\alpha_e L$ fall further away from the bandwidth limits due to the increased level of reabsorption events.

Furthermore, a series of devices with different concentrations was simulated ($\Phi = 0.65$, $W/L = 2$, $L = 2$ cm) at a range of concentrations with the same mutli-exponential decay path as described for the $CsPbBr_3$ nanocrystals, demonstrated in Figure S2c. Using a fourier transform, the Bandwidth of the devices was calculated and, as shown in Figure S4b, all devices fall below the limits for multi-exponential decays as described previously.

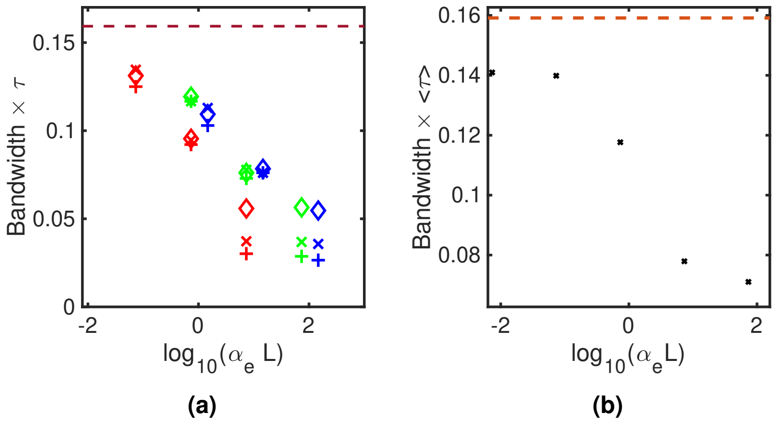

Figure S4. a) Simulated $B \times \tau$ plotted against $log10(\alpha_e L)$ for 27 devices. The devices have cross-sections 1 cm $\times$1 cm and lengths, L, 2 cm (red), 20 cm (green) and 40 cm (blue). The fluorescent lifetimes τ are 5 ns ($\times$), 10 ns ($+$), 15 ns ($\diamond$). The dotted line at $B \times \tau = 0.1593$ shows the predicted limit for the quantity $B \times \tau$ for devices with no reabsorption. b) Simulated $B \times <\tau>$ plotted against $log10(\alpha_e L)$ for 5 devices at varied concentrations. The devices have cross-sections 1 cm $\times$1 cm and lengths, L, 0.5 cm. The dotted line at $B \times \tau = 0.1593$ shows the predicted limit for the quantity $B \times \tau$ for devices with no reabsorption.)

Ratios of A_l to A_{l+1}

In the main text it is stated that the relation between A_{l+1} and A_l is constant for all l as the waveguiding efficiency of a photon is independent of the number of absorption events it has undergone, l, where l does not equal 0. Here we confirm this with a series of Monte Carlo simulations. Figure S5, shows the simulated ratios of A_{l+1} to A_l for a range of device concentrations and quantum yields, demonstrating that the ratio between values of A_l is independent of l.

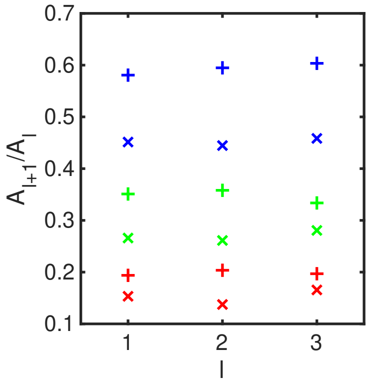

Figure S5. Values of $\frac{A_{l+1}}{A_l}$ plotted against l for a series devices with quantum yields 1 ($+$), 0.75 ($\times$), and $\alpha_e L = 10^{-0.7}$ (red), $10^{0.3}$ (green) and $10^{1.3}$ (blue). W/L for all devices is 0.04.

Equivalent Circuits

Derivation of Equivalent Circuit at Bandwidth Limits

When an LSC has no reabsorption and a fluorescence lifetime τ, it has an impulse response of $P(t) = \frac{1}{\tau}e^{-t/\tau}$ and the resulting equivalent circuit can be modelled as a simple RC circuit, shown in Figure S6. In order to derive the impulse response, we solve for V_{out}/V_{in} in the Laplace domain. In the Laplace domain, the impedance of a capacitor with capacitance C is $\frac{1}{sC}$ and impedance of a Resistor with resistance R remains R (Ref.[3]). Using the potential divider equation it can be found that:

$$\frac{V_{out}}{V_{in}} = \frac{\frac{1}{sC}}{R + \frac{1}{sC}} = \frac{1}{RC}\frac{1}{s + \frac{1}{RC}} \tag{5}$$

and as the inverse Laplace transform can be calculated as $\frac{1}{s-a} = e^{ax}$ (Ref.[4]) and substituting a for $-1/RC$ results in:

$$\frac{V_{out}}{V_{in}} = \frac{1}{RC}e^{\frac{-t}{RC}} \tag{6}$$

which takes the same form as our expected impulse response for a single-component LSC, where RC = $< \tau >$.

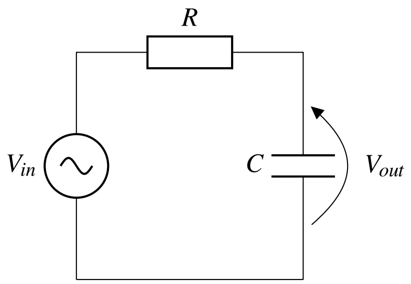

Figure S6. Equivalent Circuit for an LSC at its limits where no re-absorption occurs.

Effect of Stokes Shift

In order to explore the effects of Stokes Shift on the performance of LSC devices a series of devices were simulated ($\tau = 5$ ns, $\Phi = 0.75$, $L = 2$ cm, Cross section 1×1 cm), with varied Stokes shift. Starting with the absorption/emission spectra for $CsPbBr_3$ shown in Figure S1a, the emission spectra was red shifted, in periods of 40 nm. As expected the performance of the devices improve (bandwidth increase) with increased Stokes Shift due to decreasing overlap between absorption and emission, shown in Figure S7.

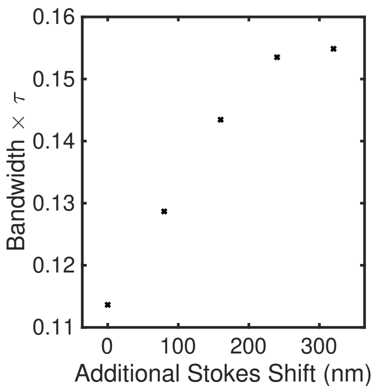

Figure S7. Simulated $B \times \tau$ plotted against increased Stokes shift plotted for 5 devices with artificially added Stokes shift. Device performance is shown to improve with increased Stokes Shift.

References

1. Tummeltshammer, C., Taylor, A., Kenyon, A. J. & Papakonstantinou, I. Losses in luminescent solar concentrators unveiled. *Sol. Energy Mater. Sol. Cells* **144**, 40–47, DOI: 10.1016/j.solmat.2015.08.008 (2016).
2. Lakowicz, J. R. *Principles of fluorescence spectroscopy* (Springer US, Boston, MA, 2006).
3. Moura, L. & Darwazeh, I. *Introduction to Linear Circuit Analysis and Modelling* (Elsevier, 2005).
4. Kirkwood, J. R. *Mathematical Physics with Partial Differential Equations* (Elsevier, 2013).
